# Acute Intestinal Obstruction Caused by Impacted Gall-Stone

**Published:** 1881-12

**Authors:** John Walters, W. A. Berridge

**Affiliations:** M. B. Lond.


### Acute Intestinal Obstruction Caused by Impacted Gall-Stone.

By John Walters, M. B. Lond., and W. A. Berridge, M. R. C. S., Etc.

Mrs. G——, widow, aged sixty-nine, had always been tolerably healthy; had suffered occasionally from bronchitis, and two years ago had an attack of acute recurring pain in the right side, called pleurisy, but no definite friction sound could be made out. She never was jaundiced; was in the habit of taking an aperient pill occasionally, and had lately become very stout. The patient was seized on the evening of February 2d, 1880, with acute abdominal pain and vomiting; her bowels had acted freely during the day. When seen about 11.30 P. M. she had just vomited a quantity of dark "bilious" fluid. Pulse 72; respiration 24; temperature 98.5°. Tongue clean and moist; urine abundant, containing lithates, but no albumen. She complained of great pain in the abdomen, especially on the right side, and kept moaning, "Oh, the pain, the pain." There was no abdominal tenderness or tympanites; no swelling or lump could be felt, and there was no external hernia. The diagnosis was, "She is passing a gall-stone." Opium was prescribed, hot fomentations and brandy, etc. On February 3d the symptoms continued; she vomited about every four hours. Before vomiting her face was sunken and distressed, and the pulse quickened to 120; after vomiting she became quite composed, and the pulse quiet. Nothing whatever passed per rectum; an enema was given and repeated, but only a very small quantity of fæces came away and a little flatus. Another enema was given on the 4th, but returned quite clear. On the 5th the vomiting began to be offensive; on the 6th it was decidedly fæcal in odor, and continued so on the 7th and 8th. During the first part of her illness she was chiefly under the care of Mr. Hallowes and Mr. Berridge. On the 6th she was seen by Dr. Holman, and opium was freely given; on the 7th matters had not mended, and it being evident that we had a case of acute intestinal obstruction to deal with, it was decided to explore the obdomen on the next day if there was no improvement. Accordingly on Feb. 8th, or the seventh day of her illness, after consultation with Drs. Holman and Walters, and Mr. Hallowes, ether was given by Mr. Skelting at 4 P. M., and the abdomen opened by Dr. Walters with full antiseptic precautions. An incision five inches long was made in the median line from the umbilicus downwards, and the hand introduced. A hard mass was found in the middle of the small intestines obstructing the bowel. The bowel was distended above the obstruction, collapsed and empty below. This was drawn to the surface, the gut opened by a longitudinal incision along its free margin, and a large impacted gall-stone removed. The wound in the bowel was closed with a continuous suture of carbolized silk, the bowel was then cleaned and returned into the abdominal cavity. The intestines were seen to be injected and their peritoneal surface roughened. The abdominal wound was closed in the ordinary way. The patient was put to bed and given an opium suppository, and hot bottles to the feet, etc. She had afterwards a little champagne and warm water to relieve flatulence. In the evening the patient seemed to have recovered from the shock of the operation and had no more vomiting. Pulse 84; respiration 24; temperature 99°. She passed a fair night, and had no pain or tympanites. The urine was drawn off by catheter. Next morning the pulse was 120; respiration 26; temperature 99°, and she appeared to be doing well. About 2 P. M. she became rather suddenly collapsed, and died about 3 P. M.

At the autopsy next day a little recent peritonitis was found. The wound in the bowel looked healthy and was quite closed; it was about the middle of the small intestine. The liver was large and fatty; no gall-bladder could be found, but where it ought to have been the duodenum was adherent to the liver by a mass of fibrous tissue. On cutting into this some small fragments of gall-stones were seen in the duct. A ragged opening existed in the upper part of the duodenum where it was adherent to the liver. The abdomen was covered by a layer of fat three inches deep. Only the obdomen was inspected.

The points to which we would draw attention are: the suddenness of the attack; the complete obstruction; the small size of the stone to give rise to such severe symptoms; the facial aspect of the patient and the character of the pulse (*a*) before, (*b*) after vomiting; the relief after the operation; and comparatively easy death.

The reason for thinking the obstruction was caused by a gall-stone were: the age of the patient; the sex, being a female; her sedentary habits and mode of life; previous history; and chiefly by excluding all other causes of acute obstruction. The stone will be in the museum of the forthcoming Congress.—*London Lancet.*

